# Evaluation of Telemetric Single-Lead Cardiac Transmitter and Apple Watch for Heart Rate Monitoring: Implications for Heart Failure Management in Home Care

**DOI:** 10.7759/cureus.80232

**Published:** 2025-03-07

**Authors:** Hideto Imai, Junichi Shimada

**Affiliations:** 1 Graduate School of Nursing for Health Care Science, Kyoto Prefectural University of Medicine, Kyoto, JPN; 2 Faculty of Nursing, Shitennoji University, Habikino, JPN

**Keywords:** apple watch, heart failure, heart rate determination, home health nursing, home medical care, wearable electronic devices

## Abstract

Objective

The study aimed to investigate the usefulness of a telemetric single-lead cardiac transmitter and the Apple Watch 7 (2021 version; Apple Inc., Cupertino, CA, USA) for monitoring heart rate during activity.

Methods

A total of 15 healthy male adults aged 24-39 years were included in this study. A wireless radio frequency electrocardiogram (RF-ECG) was used as a reference, and heart rate was measured simultaneously with the Cocoron telemetric single-lead electrocardiograph (Nipro, Osaka, Japan, NC-1BLE) and Apple Watch 7. The mean absolute error (MAE), mean absolute percentage error (MAPE), and intraclass correlation coefficient were calculated from the difference in heart rate between the RF-ECG-based wearable, Cocoron, and Apple Watch 7 at each measurement time point. Bland-Altman plots were generated.

Results

A two-way analysis of variance for the MAPE of heart rate for the Cocoron and Apple Watch 7 based on RF-ECG showed that the MAPE of the Cocoron was significantly lower than that of the Apple Watch 7 (p=0.013). This result suggests that the Cocoron provides more accurate heart rate measurements compared to the Apple Watch 7. The Bland-Altman plot revealed that the MAPE was 2.39%, 3.30%, and 3.27% for the Cocoron during supine, seated, and walking positions, respectively, and 2.26%, 3.71%, and 5.82% for the Apple Watch 7, with significantly higher values for walking compared to supine and seated. A tendency to overestimate the limits of agreement (LOA) was observed for a wider range of LOA, with the Apple Watch 7 showing a particularly large LOA upper limit of 12.02 during ambulation. This visually indicates a tendency to overestimate the heart rate during movement. This larger error is likely due to motion artifacts inherent in the wrist-based photoplethysmography (PPG) method used by the Apple Watch, which could compromise its usability in dynamic settings.

Conclusions

The Cocoron telemetric single-lead electrocardiograph transmitter measured heart rate with less error than the Apple Watch 7. During ambulation, the Apple Watch 7 had a larger error than the supine and seated positions, whereas the Cocoron had a smaller error. Since accurate and continuous heart rate monitoring is critical for the effective management of heart failure, these findings imply that devices with superior measurement accuracy, like the Cocoron, could improve clinical decision-making and patient outcomes. However, it is important to note that further studies involving heart failure patients are needed to confirm these implications in a clinical setting.

## Introduction

Heart failure patients numbered 60 million worldwide in 2017 [1], and it occurs in approximately 1-2% of the adult population in developed countries and more than 10% of those over 70 years of age [2]. The age-standardized prevalence of heart failure has shown a gradual downward trend, indicating that aging and population growth are the main causes of the increase in the number of patients [1]. In particular, elderly heart failure patients often suffer repeated relapses, making it difficult to manage the disease. In Japan, with approximately 260,000 patients hospitalized annually for heart failure [3], the number of affected patients is estimated to reach 1.3 million by 2030 [4].

Heart failure is a relatively slow and sometimes rapid disease with repeated acute exacerbations. Its prognosis is difficult to predict, with a five-year survival rate of 50% [5]. Patients are often in and out of the hospital repeatedly, and more than 20% of patients are rehospitalized within 30 days and up to 50% of patients within six months due to the exacerbation of heart failure [6]. In Japan, 24.4-29.4% of patients hospitalized for heart failure are readmitted within one year [3], making heart failure a major burden on healthcare costs.

A contributing factor to the high readmission rate is the difficulty in the self-management of heart failure in home care. Patients often notice symptoms after deterioration, making successfully tracking physiological changes at home an important component of early intervention strategies. Among these physiological factors, heart rate is important in treating and managing heart failure [7]. In patients in sinus rhythm with chronic heart failure with reduced ejection fraction (HFrEF), a heart rate greater than 70 bpm has been shown to increase the risk of hospitalization, and one that is greater than 75 bpm increases the risk of cardiovascular death [8,9]. The results of this study indicate that maintaining a heart rate above 70 bpm is important in the treatment of heart failure and requires continuous and accurate heart rate monitoring. While it is possible to monitor heart rate during hospitalization by constantly wearing an electrocardiogram monitor, a device that can be easily used for monitoring at home must be able to accurately measure heart rate without interfering with daily activities [10]. If heart rate is effectively monitored at home, early detection of symptom exacerbation becomes possible, enabling timely interventions that may reduce both symptom deterioration and hospital readmissions.

In recent years, the development of mobile devices equipped with physiological sensors has led to the widespread use of wearable devices that can continuously measure heart rate in real time [11]. Given that many of these devices are inexpensive and easy to use, they are attracting attention in big data research, the digital measurement of health, and the healthcare and fitness fields [11-14]. Currently, wearable wristwatch devices that measure heart rate include the Fitbit (Google, San Francisco, CA, USA), Garmin (Olathe, KS, USA), and Apple Watch series (Apple Inc., Cupertino, CA, USA) [15]. These methods use an optical technology called photoelectric volumetric pulse wave (photoplethysmography (PPG)) to measure heart rate. Of these, the Apple Watch has been recognized for its high accuracy in heart rate measurement [15]. On the other hand, the Cocoron (Nipro, Osaka, Japan, NC-1BLE) (hereafter referred to as Cocoron) [16] is a telemetric single-lead cardiac transmitter that detects and measures heart rate from the heart's electrical signals via electrodes. Cocoron is chest-worn and detects electrical signals directly, so it is expected to provide more accurate measurements. In this study, we evaluated the accuracy of continuous heart rate monitoring provided by a telemetric single-lead cardiac transmitter and the Apple Watch during various activities (lying down, sitting, and walking) in healthy men. The Apple Watch was selected for its reputation for high accuracy among wrist-worn devices. Furthermore, this study sought to explore how the results could inform heart rate monitoring strategies for heart failure patients, particularly in home settings.

## Materials and methods

Participants

The study included 15 healthy adult male subjects. The recruitment of participants was conducted through an open call, and the recruitment details were published on the web. Participants were enrolled using a recruitment form. The enrolled participants were contacted, the study was explained, and their consent was obtained. The inclusion criteria were as follows: the subjects had to be able to give their consent, be male, and be between 20 and 40 years of age with a low risk of cardiac disease. The exclusion criteria were as follows: subjects with a history of cardiac disease, those who take sleeping pills or anti-anxiety medications daily, and smokers.

Location

**Figure 1:**
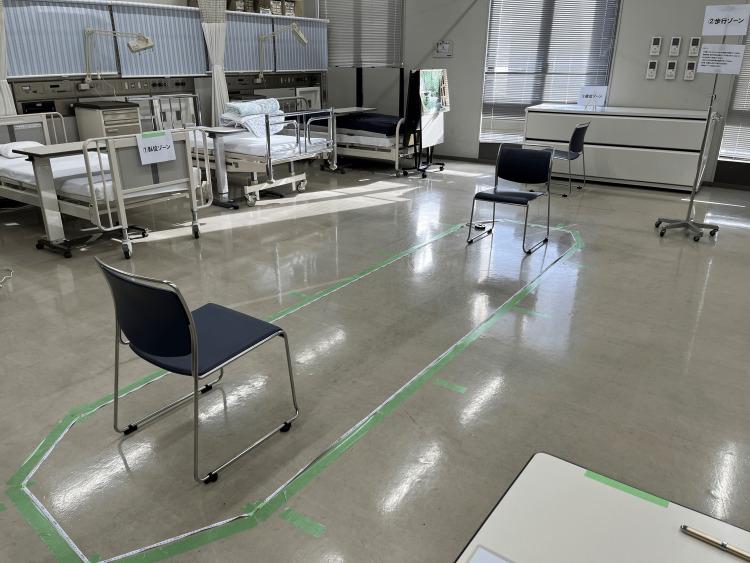
Measurement location

This study was conducted in the training room at the Kyoto Prefectural University of Medicine, Kyoto, Japan (Figure 1).

Implementation period

The study was conducted from March 11 to 25, 2023.

Survey items and methodology

Basic Attributes

Weight and body fat percentage were measured as basic attributes of the participants using the body composition meter BC-315 (Tanita Co., Ltd., Tokyo, Japan), and age, height, and occupation were investigated using a self-administered questionnaire. Although a questionnaire was administered as part of the study, its results were not included in this manuscript.

Heart Rate

Heart rate was measured using three devices simultaneously: (a) telemetric cardiac transmitter, (b) the wrist-type wearable device, and (c) high-frequency electrocardiogram wireless vital sensor (GMS, Tokyo, Japan, radio frequency electrocardiogram (RF-ECG)). The time of the three types of devices was synchronized, and participants' total heart rates were recorded for 40 minutes, which consisted of 30 minutes spent in the resting lying down position, five minutes spent walking, and five minutes spent in the resting sitting position. This protocol was designed to simulate a range of daily activities, from resting to light physical activity, which are representative of typical heart rate monitoring scenarios in everyday life.

Telemetric single-lead cardiac transmitter: Launched in 2020 in Japan, Cocoron is a telemetric single-lead cardiac transmitter [16]. It is a single-induction electrocardiograph with three electrodes. It is worn on the left anterior chest and can be used to measure the electrocardiogram waveforms and heart rate. It is cordless and weighs 20 grams. As its data can be obtained in comma-separated value (CSV) format using the compatible smartphone application "Cocoron," continuous measurement is possible while the device is worn. The heart rate data acquisition interval was set to five seconds, and heart rate was measured.

Wrist-type wearable device: The Apple Watch 7 (2021 version; Apple Inc., Cupertino, CA, USA) measures heartbeats using PPG [14]. Data were obtained from the iPhone Health Care app in Extensible Markup Language (XML) format. The timing of heart rate data acquisition could not be arbitrarily set. The inability to set precise data acquisition timings on the Apple Watch may result in irregular sampling intervals and introduce systematic bias, potentially affecting the accuracy and comparability of its heart rate measurements. The "indoor walking" mode was selected in the Apple Watch Workout app to measure heart rate while the participant was sitting or walking [12]. When the "indoor walking" mode was selected, heart rate could be obtained approximately once every five seconds.

High-frequency electrocardiography wireless vital sensor: The high-frequency electrocardiogram wireless vital sensor (hereafter referred to as RF-ECG) is an electrocardiograph used in research [17]. It is a 10 g wireless sensor. It is comprised of a wireless transmitter worn around the pericardial area and a Universal Serial Bus (USB) receiver connected to a laptop. Data were obtained in CSV format using MemCalc/Bonaly Light real-time analysis software (GMS Co., Ltd, Tokyo, Japan). After the electrodes were attached, we checked thoroughly to make sure that the electrocardiogram waveform was being properly displayed on the computer. While the data was being collected, the researchers constantly monitored to make sure that the electrocardiogram waveform and heart rate were being obtained and worked to ensure accuracy.

Procedure

**Figure 2:**
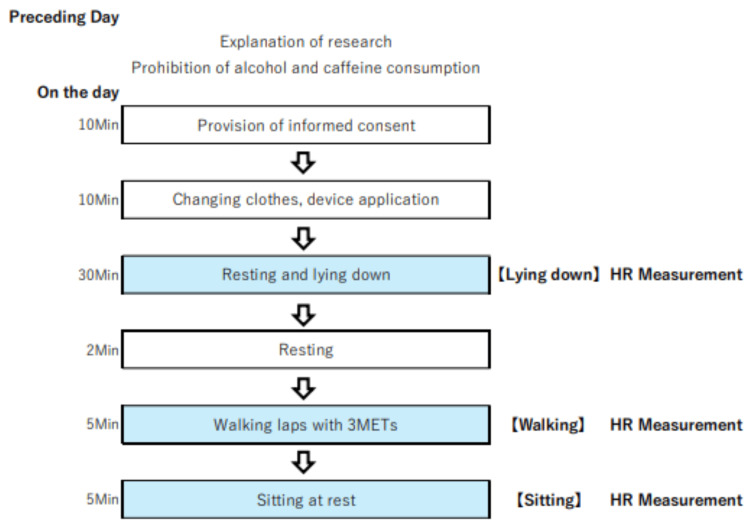
Study procedures METs: metabolic equivalent of task; HR: heart rate

The experimental procedure is illustrated in Figure 2. The room temperature was set between 20°C and 22°C. By conducting the study indoors in a temperature-controlled environment, the effects of seasonal temperature fluctuations were eliminated. The participants refrained from consuming alcohol and caffeine the previous day. Just before the measurement started, the participants were informed about the study again in writing and consented to participate; this process took approximately 10 minutes. The experiment lasted for approximately 50 minutes and consisted of the following steps: After changing into the examination clothes, the subjects were fitted with three different devices (five minutes for changing clothes and three minutes for fitting the devices). A loose-fitting examination gown that does not constrict the body was chosen to avoid interference with the measurements of each device. The Cocoron was placed on the upper part of the left chest, and the RF-ECG device was positioned below it, ensuring that they did not overlap, while the Apple Watch 7 was worn on the left wrist. Prior to electrode attachment, the skin was thoroughly wiped to remove any oils or debris, ensuring optimal contact and reducing potential artifacts. Measurements were initiated, and subjects were placed in a resting lying down position in bed for 30 minutes, followed by two minutes of rest. They then walked a 10 meter course per week for five minutes at 67 m/min (three metabolic equivalent of task (METs)), after which they maintained a resting sitting position in a chair with a backrest for five minutes. Since three METs is considered equivalent to a normal walking speed and represents an exercise load that covers many daily activities [18], the walking conditions in this study were set to assume standard walking. The walking test was conducted on a circular course set at 10 meters per lap. Participants were instructed to walk at a pace of approximately 10 seconds per lap (equivalent to about 67 m/min). To help them maintain this pace, a clock with a second hand was placed in a visible position. The experiment was completed by measuring body weight and completing a self-administered questionnaire survey, which took approximately 10 minutes.

Statistical processing

All analyses were performed using R (version 4.2.1; R Foundation for Statistical Computing, Vienna, Austria).

Assessment of Heart Rate Accuracy

The mean error (ME), mean absolute error (MAE), and mean absolute percentage error (MAPE) were used to evaluate the mistakes. Outliers can still occur during actual use and can be analyzed without removing the devices to properly assess their accuracy [14]. In this study, only the available data were analyzed, and any missing data were excluded without imputation or replacement. The synchronization method involved first adjusting the clocks of each device to the second level. Then, the timestamps of the acquired data were checked and aligned.

ME

The mean difference was calculated between the measurements from the RF-ECG and those from the Cocoron and Apple Watch 7.

MAE

The mean absolute differences between the measurements from the RF-ECG and those from the Cocoron and Apple Watch 7 were calculated.

MAPE

The MAPE for RF-ECG was calculated for the Cocoron and Apple Watch 7 by averaging the percentages of individual absolute errors. The acceptable error rate was defined as ±5%, and MAPE was used to quantify the relative error in percentage terms, making it suitable for evaluating measurement accuracy across different scales. These methods are widely used for assessing measurement accuracy in similar studies [14,15,19].

A two-way analysis of variance (ANOVA) was performed with the device (Cocoron, Apple Watch) and activity (lying down, sitting, and walking) as factors and MAPE as the dependent variable. Multiple comparison tests were performed to determine the MAPE for each device and activity.

*Bland-Altman Analysis and Limits of Agreement *(*LOA*)

The Bland-Altman analysis is a statistical method used to evaluate the agreement between two measurement techniques [20]. This approach involves calculating the mean difference (bias) and the LOA, defined as the bias±(1.96×SD), where SD is the standard deviation of the differences. By examining the bias, we can assess whether there is a systematic error (i.e., a consistent overestimation or underestimation) between the methods. Additionally, the spread of the LOA indicates the variability in the differences, providing insight into the overall consistency of the measurements. This method is the recommended method for determining agreement between medical devices, widely used for assessing measurement accuracy in similar studies. In this study, the agreement between RF-ECG, Cocoron, and Apple Watch 7 was evaluated using the Bland-Altman analysis. 

Intraclass Correlation Coefficient (ICC)

ICC analysis was performed to determine the heart rate correlation between the Cocoron and Apple Watch measurements and RF-ECG measurements. The limits of ICC were based on the suggestions of Navalta et al.'s study [19]. ICC>0.90 was considered excellent, 0.75-0.90 good, 0.60-0.75 moderate, and <0.60 low. The ICC threshold was chosen based on its use in previous studies, as it facilitates comparison between our results and those of prior research, allowing for easier interpretation and discussion within the established practices of the field.

Ethical considerations

At the time of recruitment, the purpose and content of the study were explained in writing to the subjects, and their consent to participate was obtained. After the experiment, we explained that consent could be withdrawn until data analysis started, that the participant would not suffer any disadvantage, and that all withdrawn subject data would be deleted and not be used in this study. If there was anything in the self-administered questionnaire that the participant did not want to answer, they did not have to answer. Participants were assigned a survey number so that they could not be identified. Throughout the study, participant privacy and data security were maintained by anonymizing all collected data, storing it on encrypted, password-protected servers, and restricting access to authorized personnel only. Approval for this study was obtained from the Kyoto Prefectural University of Medicine Ethics Committee (approval number: ERB-E-519).

## Results

Characteristics of participants

All 15 healthy male adults, aged 24-39 years, who participated in the study completed the survey. All participants completed the experiment on schedule. The participants' characteristics are presented in Table 1.

**Table 1:** Participant characteristics SD: standard deviation

Characteristic n=15	Value
Age (years), mean (SD)	30.1 (4.53)
Height (cm), mean (SD)	174.5 (6.76)
Weight (kg), mean (SD)	65.3 (7.89)
Body mass index (kg/m^2^), mean (SD)	21.4 (2.10)
Body fat percentage (%), mean (SD)	17.4 (3.26)

Heart rate error

A total of 13,788 pairs of heart rate data were analyzed. In total, 6,863 pairs of heart rate data from the Cocoron and 6,925 pairs of heart rate data from the Apple Watch 7 were obtained. The reason for the difference in the number of data acquired between devices was that the Apple Watch could not set the data acquisition interval at will and the number of data that could be acquired differed.

In the lying down position, MAE and MAPE were 1.49 and 2.39% for the Cocoron, while 1.41 and 2.26% for the Apple Watch. In the sitting position, MAE and MAPE were 2.21 and 3.30% for the Cocoron, while 2.55 and 3.71% for the Apple Watch. In the walking position, MAE and MAPE were 2.63 and 3.27% for the Cocoron, while 4.50 and 5.82% for the Apple Watch (Table 2).

**Table 2:** Heart rate error ME: mean error; MAE: mean absolute error; MAPE: mean absolute percentage error; ICC: intraclass correlation coefficient

	Lying down		Sitting		Walking	
	Cocoron	Apple Watch	Cocoron	Apple Watch	Cocoron	Apple Watch
ME	-0.39	0.17	-0.50	0.11	-1.12	3.08
MAE	1.49	1.41	2.21	2.55	2.63	4.50
MAPE (%)	2.39%	2.26%	3.30%	3.71%	3.27%	5.82%
ICC (95%CI)	0.97 (0.97-0.98)	0.97 (0.97-0.97)	0.96 (0.95-0.97)	0.94 (0.93-0.94)	0.94 (0.92-0.96)	0.84 (0.65-0.91)

The ICC was 0.97 (0.97-0.98) for the Cocoron and 0.97 (0.97-0.97) for the Apple Watch in the lying down position, 0.96 (0.95-0.97) for the Cocoron and 0.94 (0.93-0.94) for the Apple Watch in the sitting position, and 0.94 (0.92-0.96) for the Cocoron and 0.84 (0.65-0.91) for the Apple Watch in the walking position. The Cocoron and Apple Watch 7 showed excellent correlation in the lying down and sitting positions. During walking, the Cocoron showed an excellent correlation, and the Apple Watch showed a good correlation (Table 2).

Comparison of the Cocoron and Apple Watch 7

**Figure 3:**
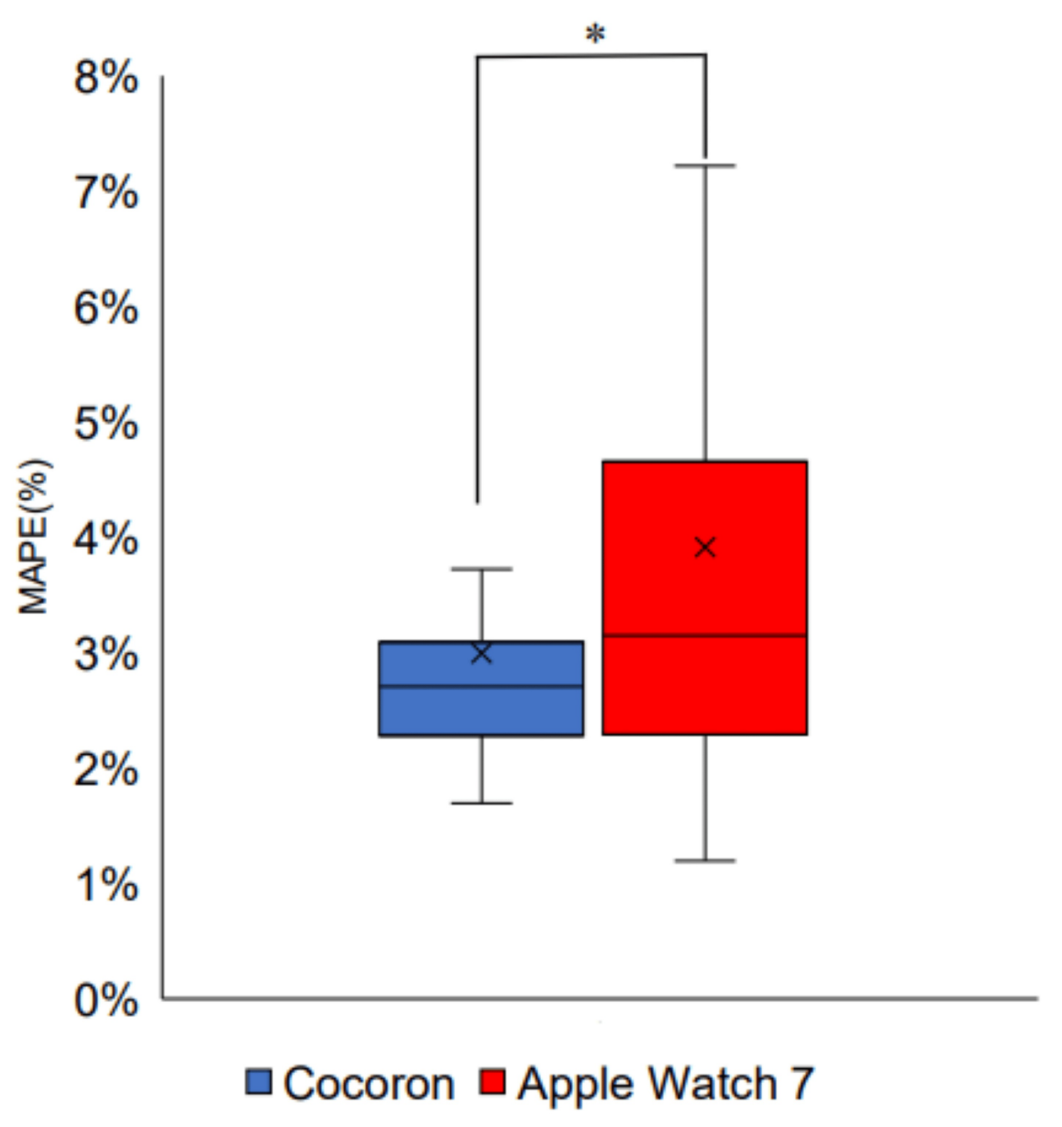
Comparison of MAPE between the Cocoron and Apple Watch 7 Two-way ANOVA (*p=0.013). ANOVA: analysis of variance; MAPE: mean absolute percentage error

**Figure 4:**
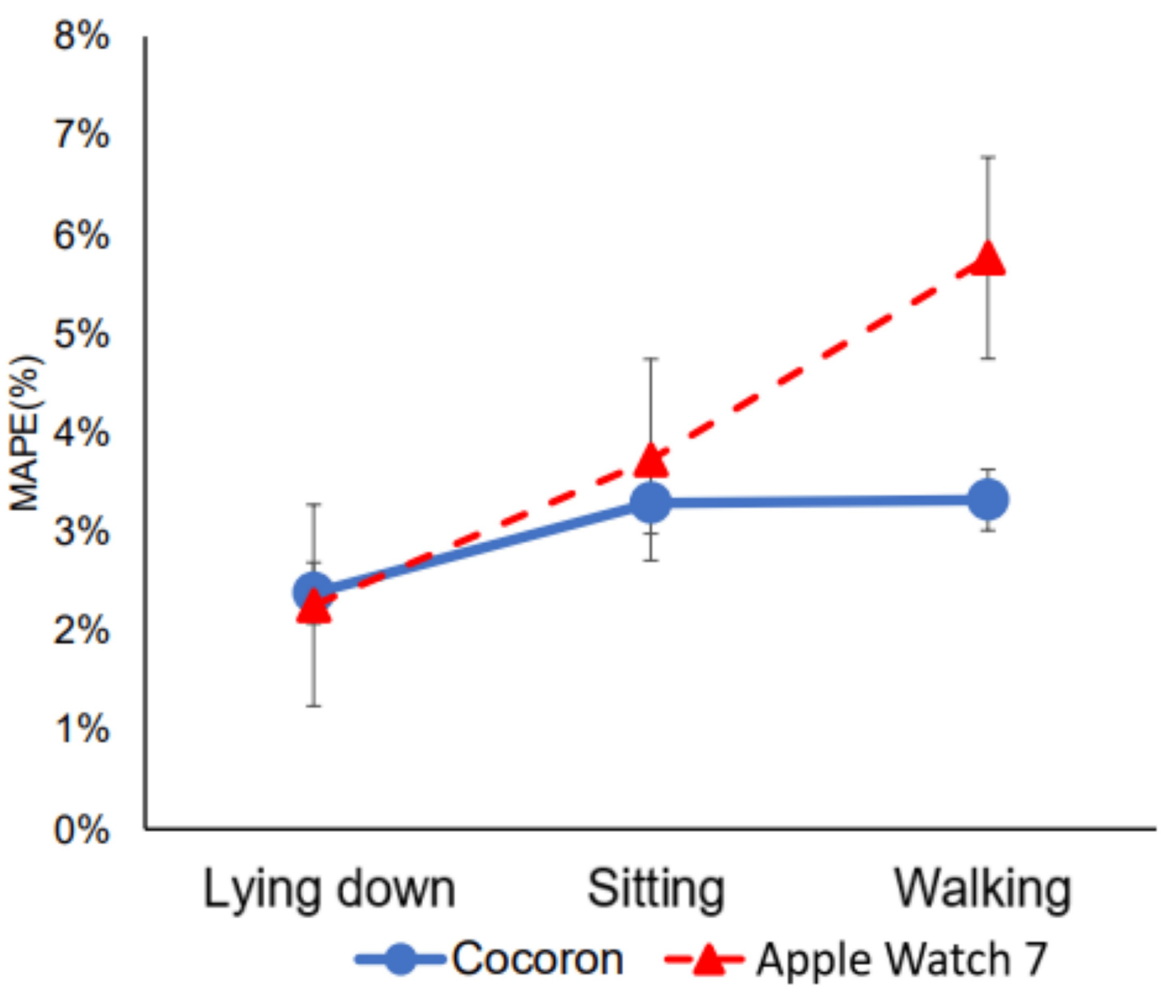
MAPE for each activity MAPE: mean absolute percentage error

A two-way ANOVA was performed using the device (Cocoron, Apple Watch)×activity (lying down, sitting, and walking)×MAPE (%) (Table 3). A main effect was found for the device (F (1, 84)=6.49; p=0.013) and activity (F (2, 84)=12.7; p<0.001). There was also an interaction between the device and activity (F (2, 84)=4.67; p=0.012); the Cocoron had a significantly smaller MAPE than the Apple Watch 7 (Figure 3). The MAPE between activities was substantially greater for the Apple Watch 7 when walking than when lying down and sitting. Simultaneously, the Cocoron showed no significant change in MAPE between the activities (Figure 4).

**Table 3:** Two-way ANOVA for MAPE Two-way ANOVA with device (Cocoron, Apple Watch) and activity (lying down, sitting, walking) as factors and MAPE as the dependent variable. ANOVA: analysis of variance; MAPE: mean absolute percentage error; df: degrees of freedom

	Sum of squares	df	F	P
Device	18.97	1	6.49	0.013
Activity	74.24	2	12.7	<0.001
Device×activity	27.3	2	4.67	0.012
Residuals	245.68	84		

Bland-Altman and LOA

**Figure 5:**
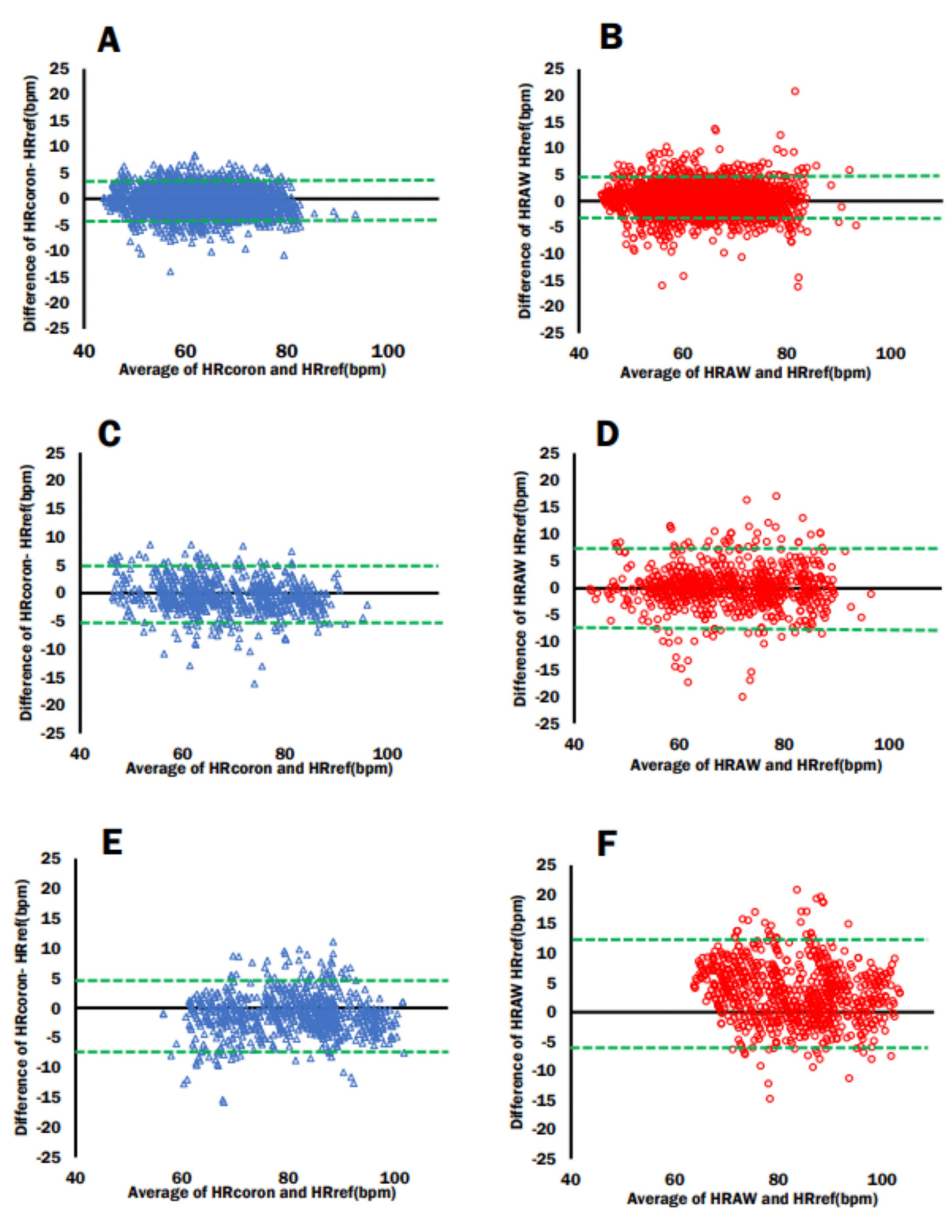
Comparing Bland-Altman plots and 95% limits of agreement for each activity (A) The Bland-Altman plot comparing the Cocoron and RF-ECG while lying down. (B) The Bland-Altman plot comparing the Apple Watch and RF-ECG while lying down. (C) The Bland-Altman plot comparing the Cocoron and RF-ECG while sitting. (D) The Bland-Altman plot comparing the Apple Watch and RF-ECG while sitting. (E) The Bland-Altman plot comparing the Cocoron and RF-ECG while walking. (F) The Bland-Altman plot comparing the Apple Watch and RF-ECG while walking. Green dotted line: limits of agreement; mean±1.96×SD. RF-ECG: radio frequency electrocardiogram; HRcoron: Cocoron-derived heart rate measurement; HRref: RF-ECG-derived heart rate measurement; HRAW: Apple Watch 7-derived heart rate measurement

In the lying down position, the Bland-Altman plot shows an ME of -0.39 with an LOA lower limit of -4.13 and upper limit of 3.3 for the Cocoron (Figure 5A), whereas it shows an ME of 0.17 with an LOA lower limit of -3.72 and upper limit of 4.09 for the Apple Watch 7 (Figure 5B). In the sitting position, the ME, LOA lower limit, and LOA upper limit were -0.5, -5.93, and 4.92, respectively, for the Cocoron (Figure 5C). In comparison, the ME, LOA lower limit, and LOA upper limit were 0.11, -6.91, and 7.12, respectively, for the Apple Watch 7 (Figure 5D). During the walking portion of the experiment, the ME, LOA lower limit, and LOA upper limit were -1.12, -7.22, and 4.97, respectively, for the Cocoron (Figure 5E), while the ME, LOA lower limit, and LOA upper limit were 3.08, -5.86, and 12.02 for the Apple Watch 7 (Figure 5F). Based on the visual inspection of the Bland-Altman plots and the ME values, it was observed that the Apple Watch tended to overestimate the heart rate compared to the RF-ECG during walking.

Bland-Altman plots and 95% LOA >75 and <75

**Figure 6:**
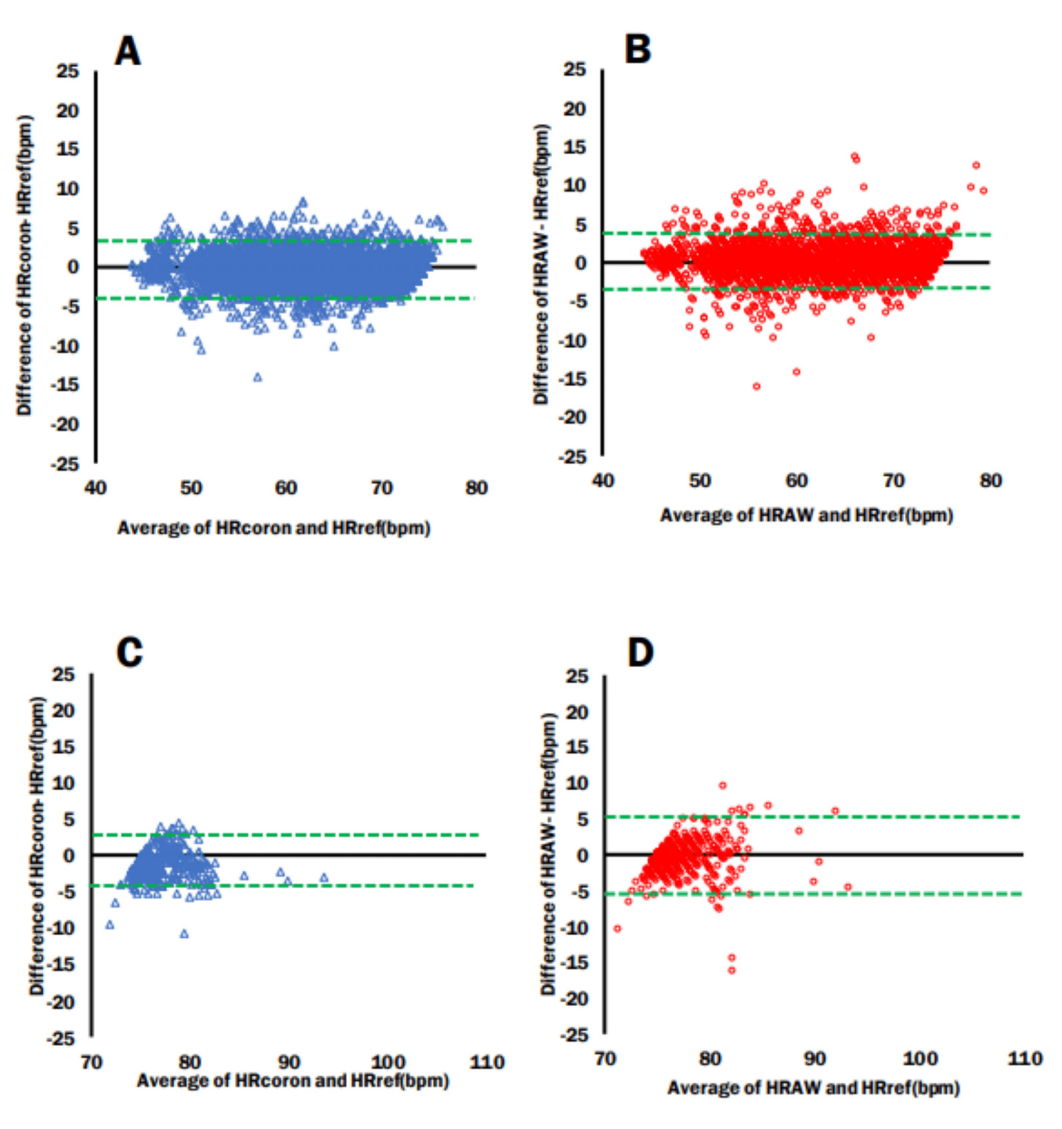
Bland-Altman plots and 95% limits of agreement: >75 bpm and <75 bpm (lying down) (A) Cocoron heart rate <75 bpm. (B) Apple Watch heart rate <75 bpm. (C) Cocoron heart rate >75 bpm. (D) Apple Watch heart rate >75 bpm. Green dotted line: limits of agreement; mean±1.96×SD. HRcoron: Cocoron-derived heart rate measurement; HRref: RF-ECG-derived heart rate measurement; HRAW: Apple Watch 7-derived heart rate measurement

**Figure 7:**
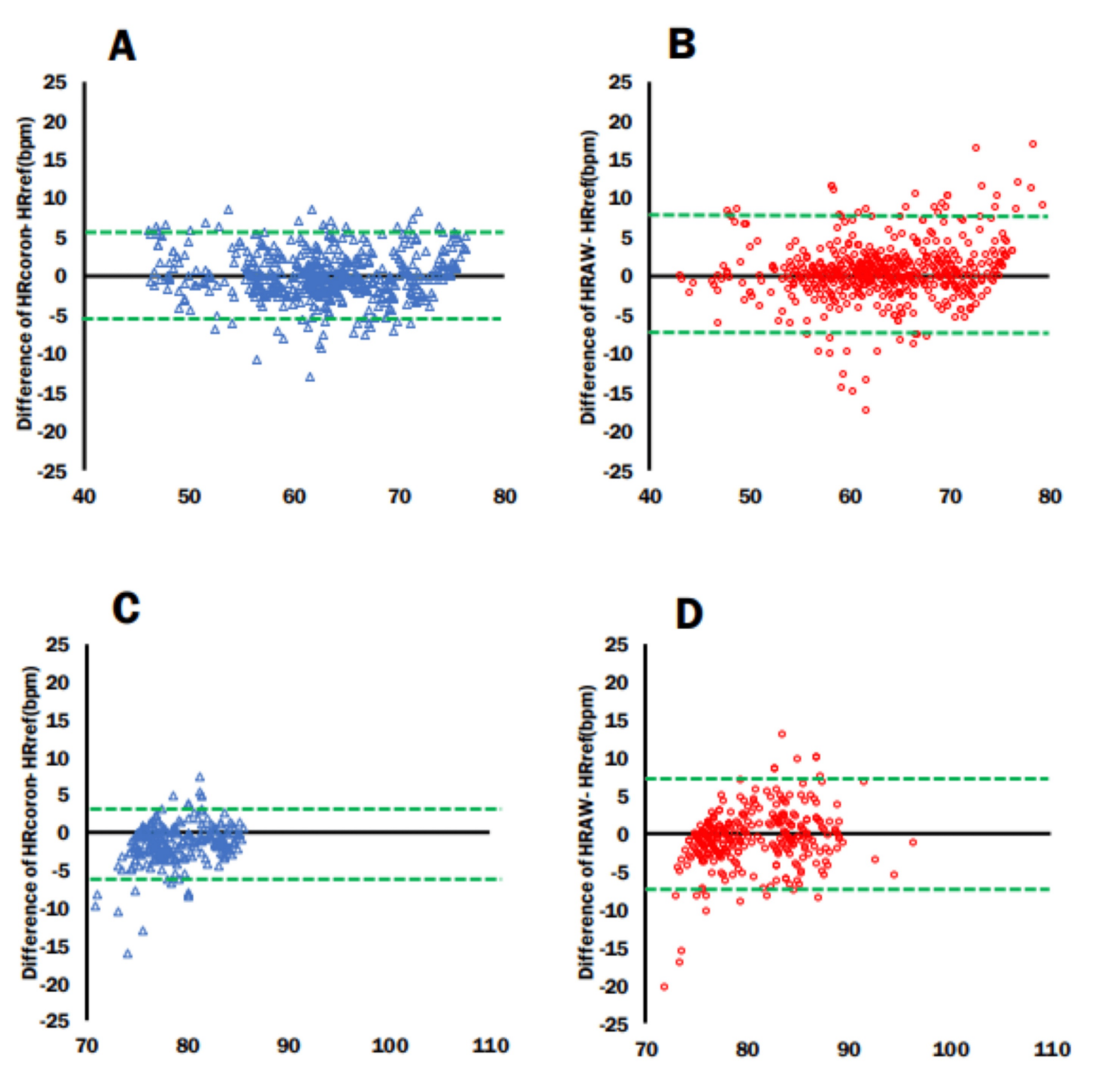
Bland-Altman plots and 95% limits of agreement: >75 bpm and <75 bpm (sitting) (A) Cocoron heart rate <75 bpm. (B) Apple Watch heart rate <75 bpm. (C) Cocoron heart rate >75 bpm. (D) Apple Watch heart rate >75 bpm. Green dotted line: limits of agreement; mean±1.96×SD. HRcoron: Cocoron-derived heart rate measurement; HRref: RF-ECG-derived heart rate measurement; HRAW: Apple Watch 7-derived heart rate measurement

Bland-Altman plots were created for the heart rates measured in the lying down and sitting positions divided by heart rates above and below 75 bpm. In heart rate management for heart failure, it is important to keep the heart rate below 75 bpm to reduce the risk of readmission and exacerbation [8,9]. Consequently, we adopted 75 bpm as the cutoff for our analysis. In the lying down position, when the heart rate was below 75 bpm, the Cocoron had an ME of -0.3, with an LOA lower limit of -4.06 and upper limit of 3.3 (Figure 6A), whereas the Apple Watch 7 had an ME, LOA lower limit, and LOA upper limit of -0.19, -3.37, and 3.76, respectively (Figure 6B). When the heart rate was above 75 bpm, the Cocoron had an ME, LOA lower limit, and LOA upper limit of -0.88, -4.52, and 2.76, respectively (Figure 6C), while the Apple Watch 7 had an ME, LOA lower limit, and LOA upper limit of 0.06, -5.3, and 5.18, respectively (Figure 6D). In the sitting position, when the heart rate was below 75 bpm, the Cocoron had an ME, LOA lower limit, and LOA upper limit of -0.01, -5.45, and 5.42, respectively (Figure 7A), while the Apple Watch 7 had an ME, LOA lower limit, and LOA upper limit of 0.46, -6.33, and 7.27, respectively (Figure 7B). Finally, when the heart rate was 75 bpm and above, the Cocoron had an ME, LOA lower limit, and LOA upper limit of -1.3, -6.01, and 3.24, respectively (Figure 7C), whereas the Apple Watch 7 had an ME, LOA lower limit, and LOA upper limit of 0.55, -7.30, and 6.19 (Figure 7D). Notably, when the heart rate exceeded 75 bpm, the width of the LOA became larger for the Apple Watch 7 in both the lying down and sitting positions, highlighting increased measurement variability under higher heart rate conditions. This tendency was more pronounced when sitting positions.

## Discussion

This study compared the accuracy of heart rate measurements during three different activity states using the Cocoron telemetric cardiac transmitter and the Apple Watch. This is the first study to evaluate the accuracy of Cocoron heart rate monitoring during activity.

Participants' attributes

In this study, several conditions were set and implemented to eliminate the factors influencing heart rate as much as possible. Since heart rate is sensitive to the female menstrual cycle and hormones, only men were included in the study [21]. Subjects with arrhythmia were excluded from the study. The study was limited to participants with no history of cardiac disease and up to 40 years of age to exclude those with arrhythmias. Individuals with a history of medication use in specific fields and smoking were excluded from the study. As a result, we were able to measure heart rate while minimizing factors that could potentially influence it. Since this study aimed to compare the accuracy of heart rate monitoring between devices, the results were obtained under highly controlled conditions. Therefore, further validation is necessary to generalize these findings. These findings have been presented in Table 1.

Accuracy of heart rate monitoring using Cocoron and Apple Watch 7

For heart rate error, a two-way ANOVA showed that the Cocoron had a significantly smaller MAPE than the Apple Watch 7 (p=0.013), indicating that the Cocoron is a more accurate device for measuring heart rate (Figure 3, Table 3).

The errors were small and good for Cocoron and Apple Watch 7 regarding lying down and sitting (Table 2, Figure 3). MAPE for the Cocoron and Apple Watch 7 devices were 2.39% and 2.26% in the lying down position and 3.30% and 3.71% in the sitting position (both ≤5%). In contrast, previous studies reported MAPE values ranging from 2.4% to 17% for the Fitbit series and from 7.87% to 24.38% for the Garmin Forerunner 225 [15,22]. Therefore, the Cocoron and Apple Watch 7 devices measured heart rate more accurately than these devices. The Cocoron had a MAPE of 3.27% for walking, and the error did not change significantly when compared to lying down and sitting positions. The Apple Watch 7 had a MAPE of 5.71% for walking, and the error was considerably greater for walking when compared to lying down and sitting (Figure 4, Table 2). In the ICC, the error was less than 0.9 for both devices in the lying down and sitting positions, while the error was 0.84 for the Apple Watch while walking, which was lower than that in the lying down and sitting positions (Table 2). As these results show, the Cocoron was able to monitor heart rate stably with minimal error, regardless of activity level. On the other hand, the Apple Watch exhibited a significantly larger error during walking. The primary reason for the observed accuracy differences between devices at walking lies in the fundamental technology and wearing positions of each device. Wrist-worn devices have been reported to be less accurate in measuring heart rate as exercise volume increases [14,23]. The Apple Watch uses PPG with green light-emitting diode (LEDs) and photodiodes to estimate heart rate based on blood flow changes. However, as movements become more pronounced, such as during walking, motion artifacts significantly affect the accuracy of PPG measurements. These artifacts arise due to variations in the contact between the sensor and the skin, external light interference, and muscle movement, all of which disrupt the consistency of light absorption and reflection [14]. As a result, wearable wristwatch-type devices become less accurate as exercise intensity increases, leading to errors in heart rate measurement. In this case, the increased arm motion during walking may have reduced the accuracy of the Apple Watch 7 heart rate measurement. In addition, the Apple Watch wristband was adjustable in size, and measurements were taken to ensure no looseness. On the other hand, the Cocoron utilizes a single-lead electrocardiogram technology to measure the heart's electrical activity directly. Because it is worn on the chest, it provides a stable measurement position and minimizes interference from movement. In addition, the Apple Watch has a disadvantage in that the timing of heart rate data acquisition is irregular and cannot be specified by the user [14]. The Cocoron also allows us to determine when to acquire data. From the prior literature, the Apple Watch is highly regarded for its heart rate measurement accuracy among other consumer wearable devices [15]. However, the Cocoron chest-mounted heart rate monitor is more accurate than the Apple Watch when used in situations that involve movement, such as during daily activities.

In the Bland-Altman plot, the Apple Watch 7 had a larger LOA and a tendency to overestimate when walking (Figure 5F). In previous studies, the dispersion of the data in the Bland-Altman plot showed that the error in heart rate measurement was larger during walking, with a slight but significant tendency to underestimate it [14,19]. Possible reasons for the differing results include differences in activity during measurement and the generational differences between Apple Watch models. The device used in this study was the Apple Watch Series 7, which is equipped with a third-generation optical sensor. In contrast, the previous study used the Apple Watch Series 3, which had a first-generation optical sensor. The differences in sensor specifications between these models may have contributed to the discrepancies in heart rate estimation observed between this study and previous research. However, the specific technical differences in sensor design and performance have not been openly disclosed by Apple. Therefore, other factors may have influenced the results rather than the characteristics of the Apple Watch [14]. With the Apple Watch, some body movements, such as walking, may increase the error and the likelihood of the heart rate being overestimated.

Due to the chronic nature of heart failure, disease recurrence and patient rehospitalization are important social health issues [24]. Additionally, an elevated heart rate can cause atherosclerosis progression in coronary arteries, diastolic dysfunction, and an imbalance between oxygen demand and supply, which can lead to ischemic heart disease [24]. In addition to heart failure, there are also reports that controlling heart rate can reduce mortality in lung cancer and gastrointestinal cancer [24]. Heart rate monitoring is important in disease management.

Downey et al. [25] reported that continuous heart rate monitoring during hospitalization resulted in better outcomes at discharge and shorter hospital stays. There is an increasing need for heart failure management at home and in the hospital [11]. The need to manage heart failure in the home is also increasing. Therefore, there is a need for wearable devices that can measure heart rate continuously and without interfering with activities of daily living, even without being present. In managing heart failure, controlling the heart rate to less than 75 bpm reduces rehospitalization rates [9]. The use of ivabradine in the treatment of chronic heart failure has also been reported to reduce rehospitalization rates. In addition, ivabradine, a chronic heart failure drug, reduced cardiovascular-related deaths (cardiovascular death) and hospitalization for worsening heart failure by decreasing heart rate [8,9]. In this study, in the lying down and sitting positions during recovery from walking, the difference in LOA in the Bland-Altman plot was greater in the lower and upper limits for the Apple Watch 7 than for the Cocoron when the heart rate was 75 bpm or higher (Figure 6, Figure 7). This trend was particularly pronounced in the sitting position, indicating that the accuracy of heart rate monitoring in the sitting position was higher with the Cocoron than with the Apple Watch when the heart rate was higher than 75 bpm (Figure 7). These findings highlight the importance of selecting highly accurate devices like the Cocoron for patients with heart failure or other chronic conditions who require precise heart rate monitoring to manage their medication and treatment plans effectively. For these patient populations, ensuring reliable heart rate data is crucial to avoid unnecessary medication adjustments and ensure effective disease management. Ivabradine is indicated for patients with a heart rate of 75 bpm or higher. Overestimating heart rate values at or above this threshold could lead to unnecessary prescriptions, potentially subjecting patients to unwarranted treatment. Therefore, in disease management, devices capable of more precise heart rate monitoring are required.

Exercise prescription for managing heart failure and diabetes requires accurate heart rate monitoring, as heart rate is used to set exercise goals and limits [26]. Moreover, cardiac rehabilitation is a comprehensive program. Cardiac rehabilitation is also central to the management and treatment of many cardiovascular diseases because it provides a structured exercise program along with secondary prevention based on comprehensive guidelines [11]. The potential use of wearable devices has been suggested for future home-based cardiac rehabilitation and in-life heart rate monitoring [27]. In this study, the Cocoron had a smaller error compared to the reference electrocardiogram, even when walking at the equivalent of three METs. In cardiac rehabilitation, the anaerobic threshold (AT) level is equivalent to three METs [18]. Activities that involve a lot of rehabilitation-like movements are likely to be more affected by motion artifacts. For this reason, wrist-type devices like the Apple Watch may produce larger errors. On the other hand, because Cocoron is chest-mounted, motion artifacts are suppressed, so it may be useful for heart rate monitoring in cardiac rehabilitation.

In the medical field, a device with low error and stable measurement is needed for rigorous disease management observation. As has been evaluated in previous studies, the Apple Watch has a small margin of error and high accuracy among wearable devices for consumers [15], but it was found that when performing activities that involve large movements, such as walking, it would take inaccurate readings and the margin of error would increase. In terms of being worn on the wrist, the Apple Watch has better usability than the Cocoron, which has electrodes attached to the chest. It is more usable than the Cocoron, which has electrodes affixed to the chest that are worn on the wrist. Therefore, while it is easy to operate and convenient to monitor the heart rate, there is a risk of large errors in real-time observation over a short period with the Apple Watch. The reason for this is that, while the error is averaged out when checking long-term heart rate trends, it can have an impact on short-term or real-time measurements. Therefore, in clinical situations where diseases are strictly managed, a device that can measure them more stably and continuously is required, making the Cocoron more useful than the Apple Watch.

As stated in "The ability to transmit data from a wearable to the clinic would erase the need for a visit, enable preventive care, and improve the quality of care that patients receive" [13], there are high expectations for the use of wearable devices in the healthcare field. Wearable devices have been reported to be effective in monitoring heart rate for the early detection of COVID-19 vaccine side effects [28]. Additionally, the growing importance of heart rate monitoring in healthcare, such as revealing the long-term effects of stress and sleep in occupations with irregular life rhythms, offers the potential for wearable devices that can continuously measure heart rate over a long period. The Apple Watch 7 has a battery capacity of only 18 hours on a full charge, while the Cocoron can be used continuously for up to 60 days. If a device cannot last a full day, users must allocate several hours for charging each time, making continuous long-term monitoring impractical. For more accurate, long-term, continuous heart rate monitoring, the chest-worn Cocoron, which allows continuous use, is beneficial.

Conventional electrocardiogram monitoring devices such as 12-lead electrocardiographs can accurately measure data, but because they can only obtain data at the time of measurement, there is a possibility that paroxysmal or exertional electrocardiogram changes will be overlooked. Many wrist-type wearable devices such as the Apple Watch are limited to only measuring heart rate over long periods of time [29]. On the other hand, although Cocoron can measure only a single lead because its electrodes are attached directly to the chest, its ability to continuously monitor electrocardiogram in everyday life makes this limitation less significant. This feature allows for the detection of cardiovascular diseases that might be overlooked when using conventional electrocardiograms, which capture data only at specific measurement points.

In recent years, there has been a lot of interest in the use of heart rate variability (HRV) to visualize the stress experienced by medical staff during treatment. When the HRV of nurses was measured continuously during surgery, the timing of stress differed between nurses who were assisting with the surgery and those who were circulating around the room [30]. Understanding the patterns of stress experienced by nurses during procedures enables countermeasures to be taken against stress, which in turn can reduce the mental burden on nurses and number of medical errors. Small, wearable electrocardiographs such as the Cocoron can measure electrocardiograms continuously without interfering with the procedures being carried out by medical staff. In the future, this data may also be harnessed to assess the stress levels of medical staff and explore practical applications aimed at reducing medical errors.

Limitations of the study

This study had limitations in sample size and demographic diversity. The participants were relatively homogeneous in terms of age, health status, and activity level, which may limit the generalizability of the findings to broader populations. Future studies with larger and more diverse participant groups are necessary to validate the results and improve their applicability to different demographic groups. This study aimed to first establish the accuracy and reliability of heart rate monitoring devices under controlled conditions before applying them to heart failure patients. Using a healthy population allowed for a more controlled evaluation of device performance without confounding factors such as medication effects, arrhythmias, or severe cardiovascular conditions. We acknowledge that further studies are necessary to validate these findings in a heart failure population. This study was conducted in a restricted laboratory setting, and only up to three METs of walking was studied. In the future, it will be necessary to compare the heart rate accuracy for various movements in daily life and examine the system's usefulness.

## Conclusions

In this study, heart rate was measured concurrently over time using a Cocoron telemetry single-lead electrocardiogram transmitter, an Apple Watch 7, and RF-ECG under three conditions: while lying down, during walking, and during the sitting recovery period following walking. The results showed that the Cocoron telemetric single-lead electrocardiogram transmitter was more accurate in monitoring heart rate than the Apple Watch 7. In particular, when walking, the measurement error was shown to be large when measured with the Apple Watch 7. The Apple Watch 7 measures using the arm, so motion artifacts were large, but the Cocoron is chest-mounted, so the measurement error was small. Under more diverse activity conditions, the error observed with the Apple Watch may become even more pronounced. Additionally, Cocoron can continuously measure heart rate for up to 60 days, demonstrating its ability to provide more accurate and sustained heart rate monitoring. The possibility of using Cocoron was suggested in situations where a device capable of strict monitoring is required when monitoring heart rate at home for the management of diseases such as heart failure. Further detailed verification is required, and in order to improve applicability to different population groups, verification with larger and more diverse groups of participants is necessary. We will also verify the accuracy and usability of heart rate monitoring under actual daily life activities.
